# LRRK2 at the interface of autophagosomes, endosomes and lysosomes

**DOI:** 10.1186/s13024-016-0140-1

**Published:** 2016-12-07

**Authors:** Dorien A. Roosen, Mark R. Cookson

**Affiliations:** 1 Cell Biology and Gene Expression Section, Laboratory of Neurogenetics, National Institute on Aging, National Institutes of Health, Bldg. 35, 35 Convent Drive, Bethesda, MD 20892-3707 USA; 2 School of Pharmacy, University of Reading, Whiteknights, Reading, RG6 6AP UK

**Keywords:** GTPases, Membrane proteins, Parkinson’s disease, Protein kinases, Vesicular trafficking

## Abstract

Over the past 20 years, substantial progress has been made in identifying the underlying genetics of Parkinson’s disease (PD). Of the known genes, LRRK2 is a major genetic contributor to PD. However, the exact function of LRRK2 remains to be elucidated. In this review, we discuss how familial forms of PD have led us to hypothesize that alterations in endomembrane trafficking play a role in the pathobiology of PD. We will discuss the major observations that have been made to elucidate the role of LRRK2 in particular, including LRRK2 animal models and high-throughput proteomics approaches. Taken together, these studies strongly support a role of LRRK2 in vesicular dynamics. We also propose that targeting these pathways may not only be beneficial for developing therapeutics for LRRK2-driven PD, but also for other familial and sporadic cases.

## Background

Understanding the etiology of a disease is often an important step for developing treatments. With many of the common neurodegenerative diseases, it is clear that single gene mutations account for some proportion of all cases while the rest are ‘sporadic’ in nature. This leads to the concept that genetic variants, acting within the context of the aging central nervous system and stochastic factors, leads to overall risk of disease. Thus, the etiology of neurodegeneration is at least partially tractable.

Parkinson’s disease (PD) falls within this rubric, in that about 10% of cases have a clear family history while the remainder are scattered throughout the population. The nature of inheritance is variable, with both dominant and recessive genes being found that have age-dependent penetrance. Furthermore, within the sporadic PD population, genome-wide association studies (GWAS) have nominated multiple genomic regions as harboring variants that contribute to overall risk of disease throughout lifetime. PD genetics is therefore rarely pure and never simple but contributes to pathogenesis and, by extension, might be leveraged for therapeutic benefit.

Here, we will focus on one specific gene for PD that is relevant for both inherited and sporadic disease that has been the subject of recent attention as a potential drug target. We will focus specifically on the underlying biology that has been uncovered in recent years to discuss the concept of pathway risk in parkinsonism.

## LRRK2 is in a pleomorphic risk locus for PD

In 2002, inherited PD in a large Japanese kindred was linked to the *PARK8* locus on chromosome 12 [1]. The same locus was found in independently ascertained families from different countries [2–4] and the underlying genetic cause, a mutation in the *LRRK2* gene, was discovered 2 years later [3, 5] an a series of *LRRK2* mutations nominated in additional families [6–10]. To date, five mutations in *LRRK2* have been shown unambiguously to segregate with familial PD and two additional variants have been nominated as risk factors (reviewed in [11, 12]). All of these *LRRK2* mutations show age-dependent incomplete penetrance, meaning that some *LRRK2* mutation carriers do not show clinical phenotypes during their lifetime [13].

Independently of mutations, GWAS approaches have also identified *LRRK2* to be a risk factor for sporadic PD [14]. The precise mechanism by which variations around the *LRRK2* gene region contribute to disease risk are not fully resolved, but given that the polymorphisms associated with sporadic PD are in the promoter region of LRRK2, a reasonable hypothesis is that these variants do not change protein structure or function but instead alter expression levels of the gene, although this remains to be formally demonstrated for LRRK2. The chromosomal region containing *LRRK2* is therefore an example of a pleomorphic risk locus, i.e. a genomic region that harbors variants that increase disease risk but by different mechanisms [15]. Additionally, *LRRK2*-driven PD is clinically indistinguishable from idiopathic PD [16]. Collectively, these observations suggest that *LRRK2* plays a general role in the etiological mechanisms of both inherited and sporadic PD.

## LRRK2 structure and enzymatic domains

**Fig. 1:**
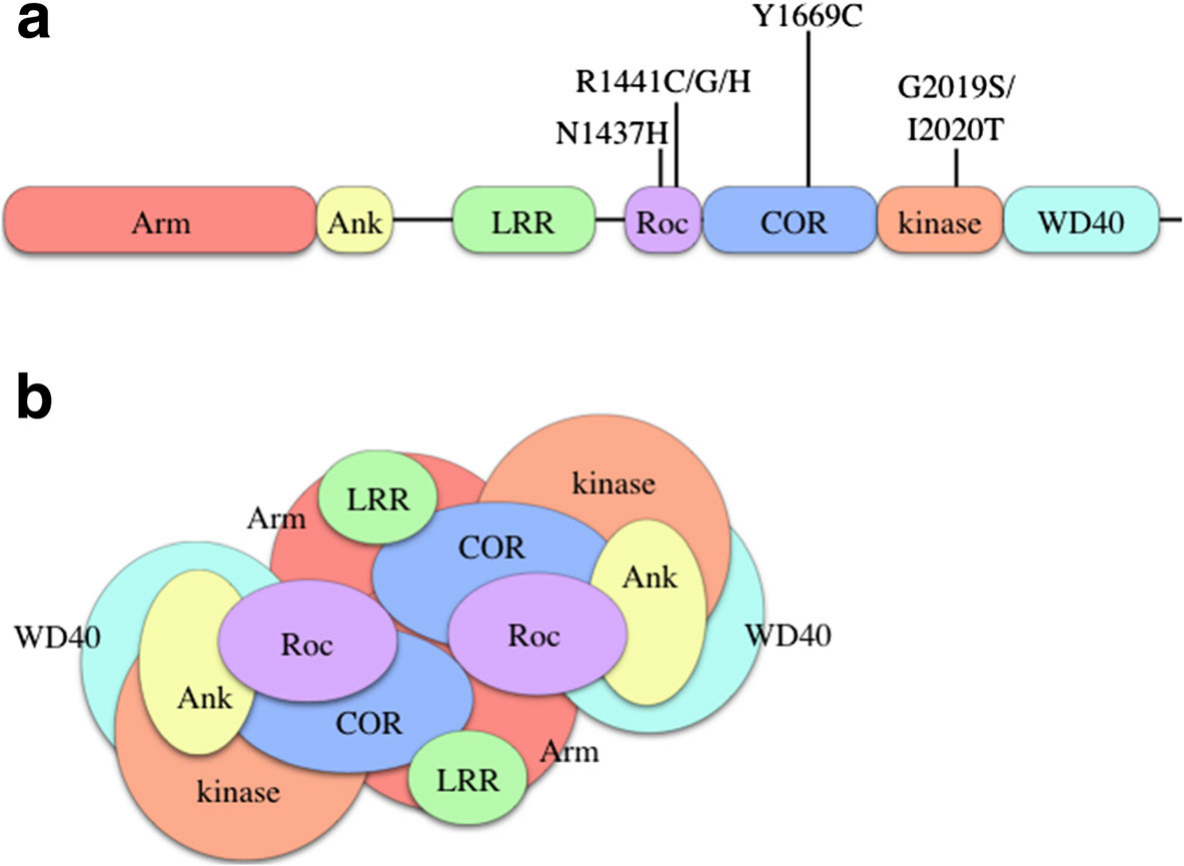
Overview of LRRK2 domain organization. **a** Linear model of the LRRK2 domains and pathogenic mutations. **b** Schematic model of homodimeric, folded LRRK2 and the approximate positioning of domains within the 3D LRRK2 structure

*LRRK2* encodes a large (2527 amino acid) multi-domain protein termed leucine rich repeat kinase 2 (LRRK2). The central portion of LRRK2 contains a Ras of Complex (Roc) GTPase and a C-terminus of Roc (COR) domain, followed immediately by a kinase domain. The ROC-COR bidomain and kinase region together constitute the catalytic core of LRRK2, which therefore encompasses two enzymatic activities. Several protein interaction domains surround this catalytic core, including N-terminal armadillo (Arm), ankyrin (Ank) and leucine rich repeat (LRR) domains and a C-terminal WD40 domain (Fig. 1). Interestingly, all the segregating mutations associated with PD are located within the enzymatic core of LRRK2 (Fig. 1) and mutated proteins have altered biochemical activity in vitro [17]. There are subtle differences between mutations, as the kinase domain mutations including G2019S and I2020T directly increase kinase activity [13] whereas those in the ROC-COR domains, the best studied of which are R1441C/G and Y1699C, decrease GTPase activity [18–21]. However, it is thought that the physical proximity of two enzyme activities encoded in the same protein structure implies that they regulate each other and lead to a co-ordinated output in cellular signaling [22, 23]. Therefore, even if mutations have differing effects on the proximal biochemical activity of LRRK2, they are likely to have a consistent effect on signaling in the cell. By extension, it is likely that evolution has selected for the multiple enzymatic and protein interaction domains of LRRK2 to be on a single polypeptide because they work together to generate one or more cellular outputs.

Despite being a large protein, several early studies showed that LRRK2 can form homodimers that localize to membrane compartments of the cell [24–26]. It is likely that dimer formation is part of the complex auto-regulatory function of LRRK2, relevant for the kinase and GTPase activities discussed above. Recently, a 3D structural model of full length LRRK2 has been described, showing that the LRRK2 homodimer adopts a compact architecture, highly suggestive of intramolecular regulation of the enzymatic activities [27]. In this model, the protein-protein interaction domains either serve to stabilize the dimer internally or are surface available for interactions with external binding partners (Fig. 1).

These biochemical and structural observations suggest, first, that LRRK2 is a co-ordinated signaling molecule that has linked enzyme activities and potentially multiple protein interaction partners and, second, that mutations associated with PD can modify these activities.

## Genetic clues for altered vesicular dynamics in PD

The next important question, is what effects LRRK2 has within cells and, therefore, within the organism. If we make the assumption that LRRK2 has some higher-level relationship with other genetic forms of PD, we might ascertain some candidates for LRRK2’s cellular role.

The first gene cloned for inherited PD was *SNCA,* which encodes a small vesicular protein abundantly expressed in the brain, α − synuclein. As for LRRK2, the genetic region surrounding *SNCA* is a pleomorphic risk locus, containing point mutations, gene multiplications and risk variants for sporadic PD. Furthermore, aggregation of insoluble α-synuclein is one of the main pathological hallmarks of PD, in the form of Lewy bodies and Lewy neurites in multiple brain regions. Because of this accumulation of protein, impaired degradation pathways have been hypothesized to be one of the underlying disease mechanisms of PD [28]. Because neurons require substantial maintenance and recycling of vesicles and their associated proteins at synapses, a particularly attractive idea is that PD might result from a failure of degradative pathways for vesicular proteins. The majority of α-synuclein is degraded through the lysosome, perhaps by a specialized process called chaperone-mediated autophagy (CMA) [29]. It is known that CMA activity diminishes with age [30] and that the protein stability of α-synuclein increases with age as well as mutations [31]. With the assumption that multiplication mutations in *SNCA* increase protein levels, a possible explanation for the age-dependent penetrance of these mutations is that the protein levels are a critical driver of toxic events in the brain.

Since the initial cloning of *SNCA*, there have been multiple PD-related genes identified that additionally converge on the related autophagy-lysosome system and vesicle trafficking pathways (summarized in Table 1, extensively reviewed in [28, 32]). We will therefore summarize some of the key characteristics and players in these intracellular events before turning to the evidence that addresses the role(s) of LRRK2 in vesicle uptake and recycling.

**Table 1:** PD-associated genes with a role in endomembrane trafficking. AD autosomal dominant, AR autosomal recessive

Gene	Inheritance	Role in endomembrane trafficking	References
Parkin	AR	Ubiquitination of damaged mitochondria for degradation by mitophagy	[86, 87]
PINK1	AR	Phosphorylation of mitochondria for parkin activation and mitophagy	[87–89]
DJ-1	AR	Mitophagy, mitochondrial dynamics	[87, 90, 91]
Fbxo7	AR	Mitophagy, interacts with parkin	[92]
α-synuclein	AD/risk factor	Substrate of CMA, pathogenic α-synuclein inhibits CMA and induces macroautophagy	[14, 28, 29, 93]
LRRK2	AD/risk factor	Autophagy, endomembrane trafficking	[14, 28]
Vps35	AD	Component of the retromer complex	[94, 95]
ATP13A2	AR	Lysosomal P5-type ATPase	[96]
DNAJC6	AR	Co-chaperone in clathrin-mediated trafficking	[97, 98]
SYNJ1	AR	Lipid phosphatase in clathrin mediated trafficking	[99]
GAK	Risk factor	Co-chaperone in clathrin-mediated trafficking, LRRK2 interactor	[14, 73]
Rab7L1	Risk factor	Small GTPase regulating endomembrane trafficking, LRRK2 interactor	[14, 73]
GBA	Risk factor	Lysosomal protease	[14, 100]
TMEM230	AD	Transmembrane protein of recycling/secretory vesicles	[101]

## The endosomal and autophagosomal pathways

**Fig. 2:**
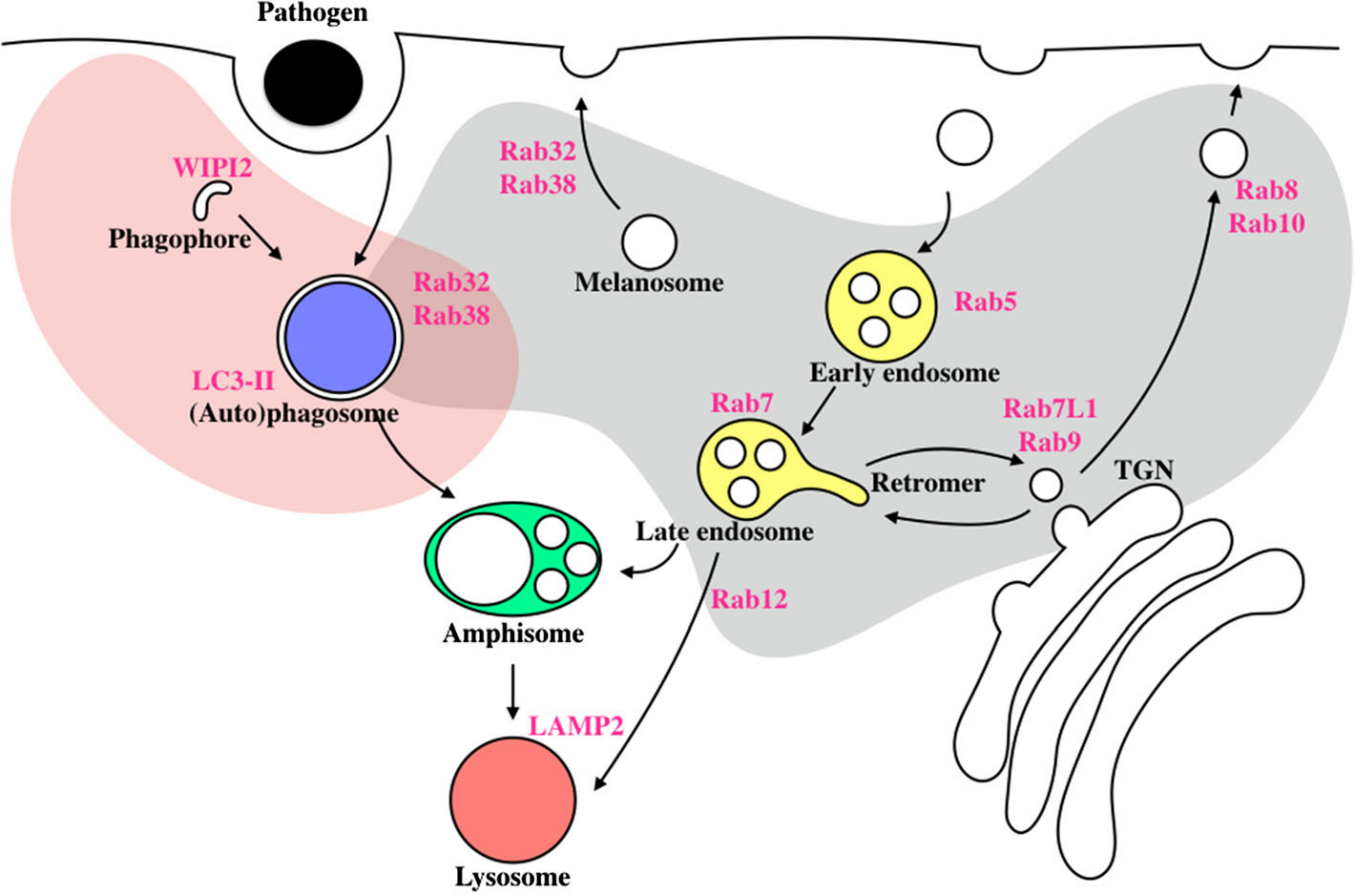
Cartoon of endosomal trafficking and macroautophagy. WIPI2 is involved in the initiation of autophagy at the phagophore. LC3-II is involved for the elongation of the autophagosomal membrane. Phagosomes are formed upon the phagocytosis of extracellular pathogens. Rab proteins, including Rab5, Rab7, Rab9, Rab7L1, Rab8, Rab10, Rab12, Rab32 and Rab38 are key regulators of endomembrane trafficking. Autophagosomes and endosomes can fuse to form amphisomes. Amphisomes on their turn fuse with lysosomes for degradation of the autophagic/endocytic cargo. *Grey* shade indicates the involvement of LRRK2 in endomembrane trafficking through physical interactors and/or kinase substrates. *Pink* shade highlights parts of endomembrane trafficking where LRRK2 is implied having a regulatory role

Two major pathways for cellular homeostasis are endocytosis and autophagy (2). During endocytosis, extracellular components are engulfed at the plasma membrane and transported and sorted via early and late endosomes [33]. The eventual destinations of endocytosed materials are varied, including rapid recycling at the post-synaptic region of neurons [34]. However, a subset of endosomes matures for subsequent fusion events with other intracellular membranous vesicles. This is a highly regulated process influenced by several cellular signaling pathways, with key involvement of the members of the Rab family of membrane-associated small GTPases [35]. Early endosomes are enriched in the signaling lipid PI(3)P, generated by the VPS34 complex. Conversion of PI(3)P to PI(3,5)P2 by the kinase PIKFyve is important for endosome maturation [36], where Rab5-positive early endosomes mature to Rab7-positive late endosomes through a transient Rab5/Rab7-postive structure [37]. Rab9 and Rab7L1 are involved in the recycling of endosomal vesicles to the trans Golgi network (TGN) via several protein complexes called the retromer [38]. Outside of endosomes, other Rabs are critical for different membrane trafficking and fusion events. Rab8 and Rab10 mediate the transport of vesicles from the TGN to the plasma membrane, whereas Rab32 and Rab38 are involved in the transport of specialized endomembrane compartments called melanosomes to the plasma membrane [39] (Fig. 2). Thus, the endosomal pathway consists of a series of discrete membrane organelles that rely on Rabs and other signaling molecules for efficient regulation.

Autophagy is derived from Greek root words for ‘self-eating’. This highly regulated process maintains cellular homeostasis through lysosomal degradation of cellular components. There are three major types of autophagy: chaperone-mediated autophagy (CMA), microautophagy and macroautophagy. During CMA, substrates are selectively but directly delivered to the lysosomes by Hsc-70 and a specific lysosomal membrane receptor, LAMP2A [30]. In microautophagy, cellular targets are directly translocated to the lysosomes but in a relatively nonselective manner that involves invagination and scission of the lysosomal membrane [40].

Macroautophagy, often referred to as simply ‘autophagy’ due to it being relatively better studied than the other two processes, involves sequestration of substrates into a specialized organelle, the autophagosome [41]. The underlying process can be broken down into 3 steps: phagophore formation, elongation of the phagophore to encircle the cargo and finally fusion of the autophagosome with lysosomes, membrane bound organelles that are enriched for proteolytic enzymes to enable degradation of their cargo (Fig. 2).

Like the endosomal system, autophagy is highly regulated by several cellular signaling pathways. In the canonical pathway, activation of the Ulk1 complex through mTOR signaling is necessary for autophagy autophagy induction. Next, the vacuolar sorting protein 34 (VPS34) complex is relocated to the phagophore for the generation of phosphatidylinositol 3 phosphate (PI(3)P). The local enrichment of PI(3)P recruits proteins associated with the initiation of autophagy, including WIPI2 [42]. Non-canonical, PI3K-independent induction of autophagy has recently been reported as well [43]. WIPI2 next functions to recruit and conjugate Atg (autophagic genes) proteins to mediate the elongation of the phagophore. In this step, the cytosolic LC3-I is cleaved and lipidated to form LC3-II on the autophagosomal membrane. This conversion of LC3-I to LC3-II is necessary for phagophore elongation to form an enclosed vesicle and is widely used as a marker for the presence of active autophagy in cells and tissues. Finally, the autophagosome fuses with lysosomes forming autolysosomes [42].

There are also specialized forms of autophagy for degradation of selective cargoes. Several organelles can be degraded after fusion with autophagosomes, for example depolarized mitochondria are cleared by mitophagy [44, 45]. In most of these cases, there are adaptor proteins that bridge the cargo to the developing autophagic membrane [46], including the general adaptor p62/sequestosome that is also often used to identify the presence of autophagy in tissues [47].

Although the above discussion outlines endosomal and autophagy as discrete pathways, in practice there is extensive cross talk between these vesicular events. For example, a subset of endosomes will fuse either directly with lysosomes in a Rab12-dependent manner or indirectly after first fusing with autophagosomes, to generate multivesicular bodies (MVBs) or amphisomes. Even more impressively, while lysosomes might be described as a waste disposal, in fact they are an important signaling platform, for example by controlling transcriptional responses to cellular metabolic state [48]. Therefore, there are likely to be signaling events that co-ordinate the overall balance between degradation and recycling of membranes and proteins in the cell.

## A physiological role for LRRK2 at vesicular membranes

The first indications for a role of LRRK2 in vesicular dynamics came from subcellular localization studies, showing localization of LRRK2 with endosomes, lysosomes and MVBs in rodent brain [49] and with punctate, vesicular structures in human brain [49, 50]. Studies in cells overexpressing low levels of tagged LRRK2 showed specific localization of LRRK2 to MVBs and autophagic vacuoles [51]. Collectively, these observations suggest that LRRK2 may have a regulatory role in the autophagic and endosomal pathways.

### LRRK2 KO models: clues for a physiological role of LRRK2 in autophagy and lysosomal function

Important evidence for a physiological role of LRRK2 in regulating autophagy came from knockout animals. Specifically, there is an accumulation of lipofuscin granules, aggregated α-synuclein and increased levels of the autophagosomal marker LC3-II in LRRK2 knockout kidneys [52]. These effects are age-dependent, in that there are bi-phasic alterations in autophagy, with an initial increase of p62 and LC3-II at 7 months and a decrease at 20 months. No changes in LC3-II were observed in an independent study of kidneys of 14 month-old LRRK2 KO mice [53].

However, no apparent signs of neurodegeneration have been observed in LRRK2 KO rodents. The 6-fold higher expression levels of LRRK2 in kidney compared to brain and the absence of its homologue LRRK1 may explain this severe kidney phenotype [52, 54]. Knockout of dLrrk, the single *Drosophila* homologue of LRRK1/2, has been shown to cause alterations in lysosomal positioning [55]. Along the same lines, knockout of the single *C elegans* homologue, Lrk-1 m causes defects in synaptic vesicle protein positioning in neurons [56].

Several studies in cells have indicated a role for LRRK2 in the regulation of autophagy. Under conditions that stimulate autophagy but prevent fusion to lysosomes, knockdown of LRRK2 led to a decreased accumulation of autophagosomes [57]. LRRK2 kinase inhibition has also been shown to increase levels of the lipdated autophagosome marker LC3-II and the adaptor protein p62 [58, 59]. Recent findings have shown that this kinase-dependent regulation of LC3 lipidation is mediated through Beclin-1 signaling but independent of mTOR/ULK1 signaling, suggesting non-canonincal regulation of autophagy [60].

There is a potential discrepancy between LC3-II levels, which generally increase with LRRK2 knockout or kinase inhibition [58, 59], and accumulation of autophagosomes, which decrease under similar conditions [57]. Iit is important to note that at steady state these two measures can be difficult to interpret in terms of overall flux through the autophagy pathway. For example, both induction of autophagy and inhibition of autophagosome clearance results in the accumulation of lipidated LC3-II. In H-4 cells, a combined treatment with a LRRK2 kinase inhibitor and bafilomycin, to block lysosomal acidification, results in an additive increase in LC3-II [58]. This suggests that LRRK2 inhibition does not block flux through the overall autophagy pathway but rather increases formation of autophagosomes. By extension, these considerations suggest that LRRK2 normally functions to block autophagosome formation.

However, even these data are complicated by the observation that, in microglial cells, knockdown of LRRK2 can decrease LC3-II formation after lysosomal inhibition [57], in contrast to increases in mice [52] and H4 cells [58, 59]. It is possible therefore that there are cell-type specific signaling events that can modulate the direction of effect of LRRK2 on autophagy markers, indicating that autophagy regulation may be a downstream consequence of LRRK2 deficiency rather than a primary event.

In addition, higher levels of lysosomal markers and the lysosomal protease cathepsin D are seen in LRRK2 knockout mouse kidneys compared to their wild type counterparts irrespective of age [54]. Similar phenotypic changes, including lipofuscin accumulation and increase in lysosomal markers have been observed in LRRK2 KO rats [61, 62]. Therefore, while influencing autophagosome formation, LRRK2 may also play a role in lysosomal maturation and/or trafficking. How these two events are related is not immediately clear and, given then age-dependence of some changes [52, 54], it remains possible that alterations in one part of the autophagy-lysosome system are compensated for by alterations in other degradative processes.

### Pathogenic mutations in LRRK2 KO affect vesicular events in vitro and in vivo

The above data show that the normal function of LRRK2 appears to be related to vesicular trafficking. Several observations in different systems further suggest that LRRK2 mutations across multiple domains of the protein also alter vesicular dynamics.

Fibroblasts derived from PD patients carrying mutations across several enzymatic domains of LRRK2 (G2019S, Y1669C, R1441C) show a diminished autophagic response to starvation, measured by LC-3 conversion, compared to control fibroblasts [63]. Cells overexpressing R1441C LRRK2 show an increase in MVBs and autophagic vacuoles [51]. Overexpression of G2019S in cells also results in an increase in autophagic vacuoles and decreased neuronal process length. Knockdown of the conserved autophagy genes LC3 and Atg7 as well as inhibition of ERK signaling reversed this effect [64]. Overexpression of wild type LRRK2 in cells has also been reported to result in an increase of autophagosomes [65].

iPSC derived dopaminergic neurons from G2019S mutation carriers show an increase of autophagic vacuoles and an accumulation of aggregated α-synuclein [66, 67]. In these cells, there were no changes in *SNCA* transcription, suggesting an impaired degradation of α-synuclein [67]. G2019S LRRK2 iPSC showed a decrease in neurite length compared to control iPSC and induction of autophagy further exacerbated this phenotype [66]. An independent study of G2019S iPSC derived dopaminergic neurons and isogenic controls also showed neurite shortening in an ERK-dependent way [67]. Notably, G2019S LRRK2-mediated effects on autophagy in cells have also been reported to be mediated through ERK signaling [68]. Finally, in vivo, mice carrying the G2019S mutation show an accumulation of autophagic vacuoles in the cerebral cortex, as do R1441C LRRK2 transgenic mice [69].

The collective data available therefore suggests that mutant forms of LRRK2 decrease LC3 lipidation and result in the accumulation of autophagic vacuoules. The observations with LC3 are consistent with the data from knockout and inhibition models that LRRK2 normal function is to block autophagosome formation and that dominant mutations enhance this activity. However, the subsequent accumulation of autophagic vesicles suggests that there are additional effects of mutations in LRRK2 on the overall function of the autophagy-lysosomal pathway. One possible explanation for this apparent discrepancy comes from the observec concurrent increase in autophagic vacuoles and accumulation of α-synuclein in cells with G2019S LRRK2 [66, 67]. Because α-synuclein is degraded by the lysosome [70], the available data could suggest that G2019S mutant of LRRK2 simultaneously block autophagosome formation and lysosomal function, which contrasts perhaps with the accumulation of lysosomal enzymes in LRRK2 knockout animals [52, 54].

### Candidate mechanisms for LRRK2 effects on vesicular trafficking

There are several potential mechanisms by which LRRK2 may affect vesicular trafficking. Indirect mechanism, such as those where LRRK2 has direct effects on metabolic or cellular signaling pathways that then indirectly affect autophagy, may explain some of the observed correlated changes noted above. However, here we will focus on regulation of vesicular trafficking events that are potentially mediated by direct protein-protein interactions. The rationale for this limitation on discussion of mechanisms is that as LRRK2 has multiple protein interaction domains, these are likely important effectors of its function in cells.

Unbiased proteomics approaches have provided important insights into the functional roles of LRRK2. Rab5 was first found to interact with LRRK2 using a yeast-two-hybrid screening approach [71]. Conversely, LRRK2 was identified as an interaction partner in a yeast-two-hybrid screen for Rab32 [72]. High-throughput protein-protein interaction arrays have shown that LRRK2 physically interacts with Rab7L1 (also known as Rab29) [73]. In the latter case, we have found that Rab7L1/Rab29 is important for recruiting LRRK2 to the TGN, along with the clathrin-uncoating protein cyclin-G associated kinase (GAK) and the co-chaperone BAG5. This protein complex may be conserved as similar proteins are important for the recruitment of Lrk-1 to the golgi apparatus in *C elegans* [74], Importantly, Rab7L1 and GAK are nominated to be risk factors for sporadic PD [14]. Clearance of Golgi-derived vesicles by the LRRK2 complex including Rab7L1 is enhanced by mutations across all enzymatic domains of LRRK2 whereas hypothesis testing LRRK2 mutations, including those that are kinase dead or cannot bind GDP/GTP, were ineffective in TGN vesicle clearance [73]. This suggests that enzymatic activities of LRRK2 are required to promote TGN clustering and clearance and that pathogenic mutations result in a gain-of-function that enhance this phenotype [73].

In addition, LRRK2 was shown to interact with a number of other Rab GTPases, including Rab32 and Rab38 [72]. Recently, phosphoproteomic screens were performed in an effort to identify *bona fide* LRRK2 kinase substrates [75]. Two screens were performed using cells from mice engineered to have either the kinase hyperactive G2019S or kinase inhibitor resistant A2016T LRRK2, in combination with treatment of distinct LRRK2 kinase inhibitors. Overlap of these screens resulted in the identification of a single LRRK2 kinase substrate, Rab10. Further analysis in HEK293FT cells indicated that Rab10 as well as Rab8 and Rab12 are direct physiological LRRK2 substrates [75].

Although publication of independent confirmation of these findings is still awaited, they suggest that one of the key functions of LRRK2, kinase activity, is important in control of Rabs and, hence vesicular trafficking events. Furthermore, in cells (but not in vitro), mutations in several different regions of LRRK2 consistently result in increased Rab phosphorylation, supporting the contention that different LRRK2 domains work together to produce functional output [75]. Along the same lines, all pathogenic mutations in LRRK2 increase Rab7L1-dependent retention at the TGN [73]. However, the precise mechanism(s) by which LRRK2 domains interact in cells remain to be determined.

Collectively, these data place LRRK2 at the scene of the crime for vesicle sorting. A recent computational analysis of the LRRK2 interactome further supports a potential role for LRRK2 in vesicular dynamics such as endocytosis and autophagy [76]. However, the range of Rabs identified suggests multiple roles for LRRK2 at different intracellular membranes. It is also of interest that LRRK2 has a different set of Rabs that appear to be direct substrates from those that were nominated as stronger binding partners, perhaps suggesting that depending on the Rab, LRRK2 may have different modes of action. Further confirmation of the binding and phosphorylation events are needed before we can be certain of the precise role that LRRK2 plays in Rab biology and vice-versa. Nonetheless, because Rab proteins are important in vesicular dynamics, these results suggest that the mechanism by which LRRK2 affects intracellular membranes is mediated via Rab interactions.

There are several pieces of evidence to suggest that, in different tissues and systems, the physiological interaction with Rabs is important for mediating the effects of mutations in LRRK2 on membrane trafficking. As well as causing changes in autophagy, pathogenic LRRK2 mutations have also been shown to lead to alterations in synaptic vesicle trafficking in neurons. Rab5 has a particularly strong role in synaptic vesicle endocytosis. Overexpression of WT LRRK2 impaired synaptic vesicle endocytosis and this effect was further enhanced by overexpression of G2019S LRRK2, whereas-expression of Rab5 rescued this phenotype [77].

Further supporting the idea that LRRK2 and Rabs co-operate to modulate vesicular trafficking, Rab7L1 KO mice have the same lysosomal pathology in the kidneys as LRRK2 KO mice and the combined deficiency of both proteins also results in a similar phenotype suggesting a genetic interaction with consistent direction between these two proteins [78]. Whether this is true for other Rabs that are direct substrates of LRRK2 is not known, and future studies are required to further substantiate the relationship between LRRK2, Rabs and regulation of the autophagy-lysosome system.

Studies in *C. elegans* neurons suggest that suggests that the LRRK2 nematode ortholog acts downstream of Rab7L1 ortholog in endo-lysosomal trafficking. Furthermore, cellular work showed that LRRK2 interacts with AP-3 as a downstream effector, essential for trafficking of lysosomal membrane proteins from the Golgi to the lysosomes [78]. The *Drosophila* homolog of LRRK2 (dLrrk) colocalizes with endosomes and lysosomes and interacts late endosomal protein Rab7. dLrrk loss-of-function mutants have abnormalities in the endosome and dLrrk can negatively regulate Rab7-dependent perinuclear localization of lysosome [55]. In contrast, a mutation in dLrrk corresponding to the G2019S mutation in LRRK2 promotes Rab7-dependent perinuclear positioning of lysosomes [55]. Accumulation of autophagosomes and presence of enlarged lysosomes and endosomes were also observed in *dLrrk* loss-of-function mutants [79]. This phenotype was rescued by overexpression of Rab9, which promotes recycling of endosomes to the TGN via the retromer, again possibly due to a direct interaction [79]. As noted above, dLrrk is paralog of LRRK1/LRRK2 [80] and therefore may interact with a slightly different or broader set of Rabs than LRRK2. Nonetheless, these collective data strongly suggest that the effects of LRRK2 across several species depend on Rab GTPases in different tissues and cells, not just in neurons.

Fibroblasts of PD patients carrying the G2019S mutation showed decreased Rab7 activity. Overexpression of G2019S as well as R1441C LRRK2 cause a decrease of Rab7 activity in cells [81]. Moreover, expression of mutant LRRK2 caused a delay in early to late endosomal trafficking, as evidenced by a decreased Rab5 to Rab7 transitioning [81]. A dramatic delay of trafficking out of late endosomes was observed in cells overexpressing G2019S and R1441C LRRK2. These late endosomes showed a marked increase in Rab7-positive tubules [81].

However, in addition to Rab proteins, LRRK2 may also mechanistically alter membrane dynamics via other important interacting proteins. LRRK2 has been shown to interact and colocalize with Sec16, a key protein involved in ER-Golgi transport [82]. The R1441C LRRK2 mutation impaired this interaction and mouse primary fibroblasts from R1441C transgenic mice showed impaired ER to Golgi trafficking [82].

LRRK2 and its *Drosophila* homologue dLRRK were shown to phosphorylate the synaptic vesicle endocytosis protein endophilin-A in vitro [83, 84]. In *Drosophila*, increased endophilinA phosphorylation by G2019S dLrrk resulted impaired synaptic endocytosis [83]. Moreover, dLRRK-dependent phosphorylation of endophilinA was recently shown to stimulate autophagy in at Drosophila synapses, highlighting cross-talk between endosomal and autophagosomal signaling networks [85].

Collectively, these data show that LRRK2 can interact with multiple vesicle-associated proteins. One of the most important remaining questions for LRRK2 biology is how binding to Rabs or other proteins influences the observed alterations in autophagy and lysosomal markers seen in cells and animal models, or whether other mechanisms are at play. A particular complexity of vesicular trafficking is that events are often inter-related as, for example, multiple Rabs co-operate to influence overall protein and vesicle sorting [35]. Thus, overall flux through a pathway may depend on interactions between multiple partners some of which may anatogonize each other. Further complicating interpretation, presumably most tissues and cells have compensatory mechanisms that will at least partially recover function in vesicle sorting. It will therefore be important to examine multiple steps of vesicular sorting to see which are consistently and directly affected by LRRK2 deficiency and mutations to determine which events are direct and which are consequential.

## Conclusions

A substantial amount of evidence shows that LRRK2 plays an important role in vesicular trafficking. LRRK2 KO models and studies using LRRK2 kinase inhibitors have highlighted a regulatory role for LRRK2 in autophagy. Proteomics approaches have greatly helped to identify physical interactors as well as *bona fide* kinase substrates of LRRK2. Importantly, given the high interconnectivity of endosomal, lysosomal and autophagosomal pathways, dysfunctions in one system may well trigger alterations in another.

However, how altered vesicular trafficking can ultimately lead to neurodegeneration is not well understood in the context of LRRK2 mutations. Understanding such pathobiological roles of LRRK2 is critical for the development of therapeutic strategies. If LRRK2 mutations result in a gain of biochemical function, targeting the kinase and/or GTPase activity of LRRK2 could be helpful to modulate disease progression. More broadly, if it is true that multiple PD-related genes converge on vesicular trafficking pathways, regulatory and partially redundant mechanisms for autophagy might be targetable for therapeutics.

## Abbreviations

AD: Autosomal dominant
Ank: Ankyrin
AR: Autosomal recessive
Arm: Armadillo, Atg, Autophagic genes
CMA: Chaperone-mediated autophagy
COR: C-terminus of Roc
LRR: Leucine rich repeat
LRRK1/2: Leucine rich repeat kinase 1/2
MVB: Multivesicular body
PD: Parkinson’s disease
PI(3)P: Phosphatidylinositol 3-phosphate
PI(3,5)P2: Phosphatidylinositol 3,5-biphosphate
Roc: Ras of complex
TGN: Trans-golgi network
Vps: Vacuolar sorting protein
